# Diffuse intrapulmonary mesothelioma mimicking pulmonary lepidic adenocarcinoma: a rare case report and review of the literature

**DOI:** 10.1186/s13000-023-01327-7

**Published:** 2023-05-16

**Authors:** Wang RanYue, Wu ChunYan, Hou Likun, Zhang LiPing, Lin JieLu, Dong ZhengWei

**Affiliations:** Department of Pathology, Shanghai Pulmonary Hospital, Tongji University School of Medicine, 507, Zhengmin Road, Shanghai, People’s Republic of China

**Keywords:** Mesothelioma, Diffuse intrapulmonary mesothelioma, Pleura, Adenocarcinoma, Lepidic, Lung cancer, Lung nodule

## Abstract

Mesothelioma, with various clinical manifestations, radiological features, and histomorphological types, can be divided into epithelioid, sarcomatoid, and biphasic types, according to their histomorphological characteristics. There is a rare growth pattern of pleural mesothelioma: diffuse intrapulmonary mesothelioma (DIM), with a distinctive pattern of predominantly intrapulmonary growth, has no or minimal pleural involvement, and simulates interstitial lung disease(ILD) clinically and radiologically. A 59-year-old man presented to the hospital with recurrent pleural effusions for 4 years and a history of asbestos exposure. Computed tomography (CT) showed bilateral pure ground-glass opacity lesions, and the tumor cells showed a lepidic growth pattern pathologically. Immunohistochemical staining was positive for CK, WT-1, calretinin, D2-40, CK5/6, and Claudin4, while TTF-1, CEA, EMA, CK7, CK20, and other epithelial markers were negative. BAP1 loss its expression, and MTAP was positive in cytoplasm. CDKN2A was negative tested by Fluorescence in situ hybridization (FISH). The final diagnosis was DIM. In conclusion, we should recognize this rare disease to avoid misdiagnosis and delayed treatment.

## Introduction

Mesothelioma is a highly aggressive malignant tumor originating from mesothelial cells, and is associated with a history of asbestos exposure or thoracic radiation therapy. Mesothelioma, which usually arises from the pleura and peritoneum, can also stem from the pericardium, testis, and female reproductive system, and rarely from the lungs. Pleural mesothelioma (PM) usually manifests as diffuse pleural thickening or multiple nodules on thoracic imaging, with or without pleural effusion. In 21% of all cases of PM approximately, the tumor may invade the pulmonary parenchyma [1]. Diffuse intrapulmonary mesothelioma (DIM), which has a predominantly intrapulmonary growth pattern, accounts for about 0.5% of all PM cases [2]. In terms of immunohistochemistry, DIM is positive for mesothelial markers and negative for BAP1, which is similar to mesothelioma. We report a rare case of DIM presenting with lepidic growth pattern, and review so far published reports summarizing their clinical and pathological characteristics.

## Case report

### Clinical summary

**Fig. 1:**
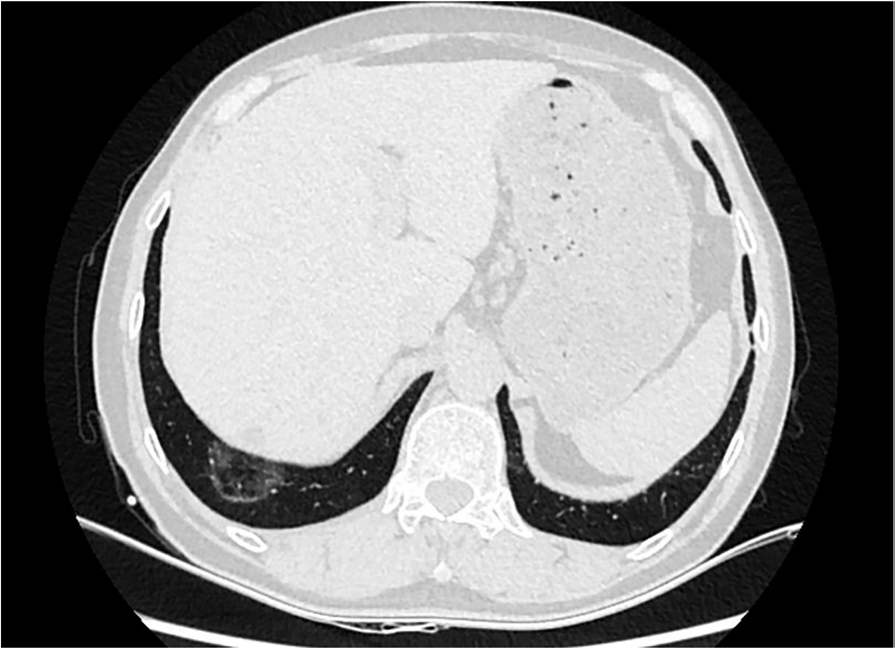
Computed tomography showed ground-glass lesions in the lower lobe of the right lung

We report the case of a 59-year-old man who complained of recurrent pleural effusion for 4 years. The patient had worked on high-temperature stoves for more than 10 years and had been exposed to asbestos-containing thermal insulation materials daily during this period. On physical examination in 2017, the patient was found to have a right pleural effusion and was not taken seriously. Computed tomography (CT) of the patient in 2019 showed patchy opacities in both lungs and pleural effusion on the right side. The pleural effusion was drained and the cytopathological report showed no malignancy. The patient was considered to have tuberculous pleurisyhad and received diagnostic anti-tuberculosis treatment for 1 month, but the result was unsatisfactory. In June 2021, the patient underwent pleural effusion drainage in another hospital due to increased pleural effusion again, and the cytopathology report showed proliferating mesothelial cells. In October 2021, the patient's right pleural effusion increased. CT showed ground-glass lesions in both lungs; the larger lesion was located in the lower lobe of the right lung, with a length of 35 mm (Fig. 1), multiple small nodules in the pleura, and no abnormal enlargement of multiple lymph nodes in the mediastinum. Laboratory examination showed that the GM test was 1.24ug/L, and the total protein in the pleural fluid was 33.0 g/L.

The patient visited our hospital for drainage of the pleural effusion. Cytology of the pleural effusion showed that the cells were small clusters or single scattered, round cells, medium size, and had a slightly higher nuclear-to-cytoplasmic ratio. A small number of lymphocytes, histiocytes, and psammoma bodies in the background. The cytopathological report suggested atypical cells.

**Fig. 2:**
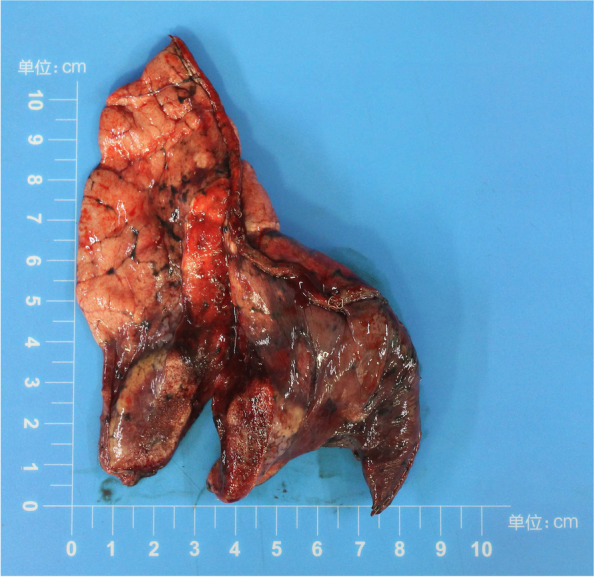
The mass in the lower lobe of the right lung, close to the visceral pleura, gray-yellow-gray-red, soft, with a clear boundary

After assessing the indications for surgery, the patient underwent a right lower lobe wedge resection and visceral pleurectomy. During the operation, the surgeon found a mass in the lower lobe of the right lung, adjacent to the visceral pleura. The visceral pleura and parietal pleura were scattered with small white nodules. The postoperative specimen showed that the maximum diameter of the mass was 33 mm, gray-yellow-gray-red, close to the visceral pleura, soft, with a local gritty feeling and a clear boundary (Fig. 2). In addition, several gray-white nodules were found on the surface of the pleura, with a diameter of 3 mm-8 mm.

### Pathologic analyses

**Fig. 3:**
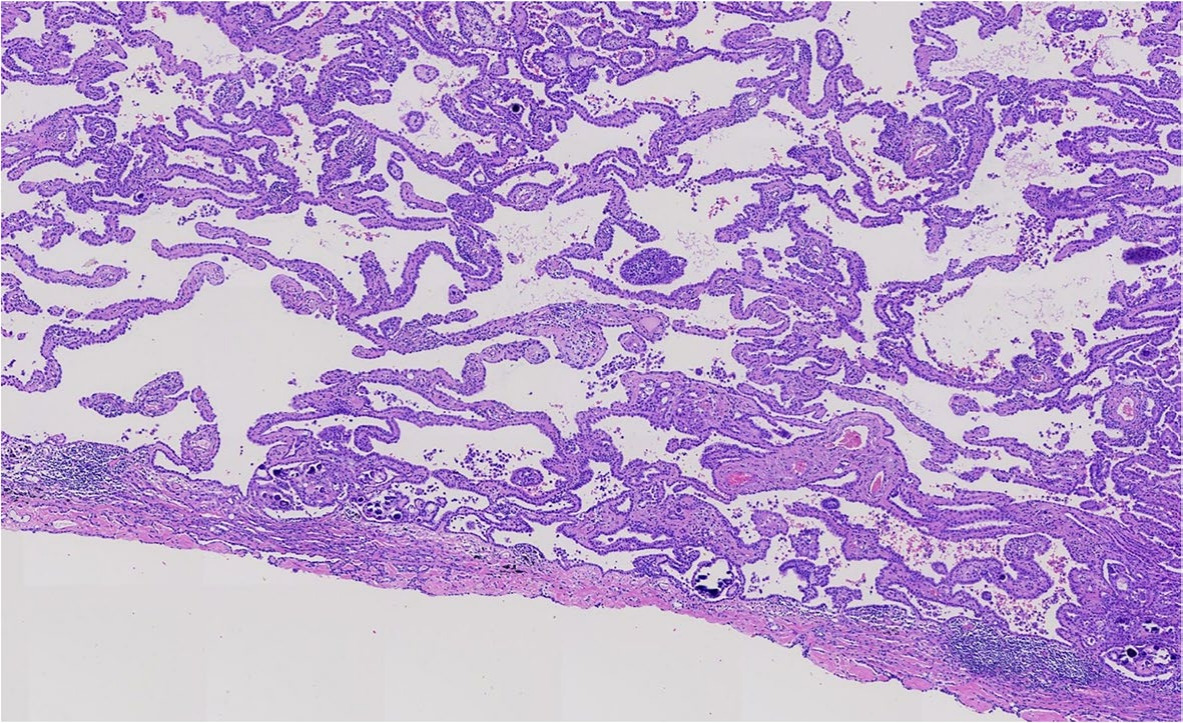
The alveolar structure exists, and the tumor grows mainly along the alveolar wall in an adherent manner

**Fig. 4:**
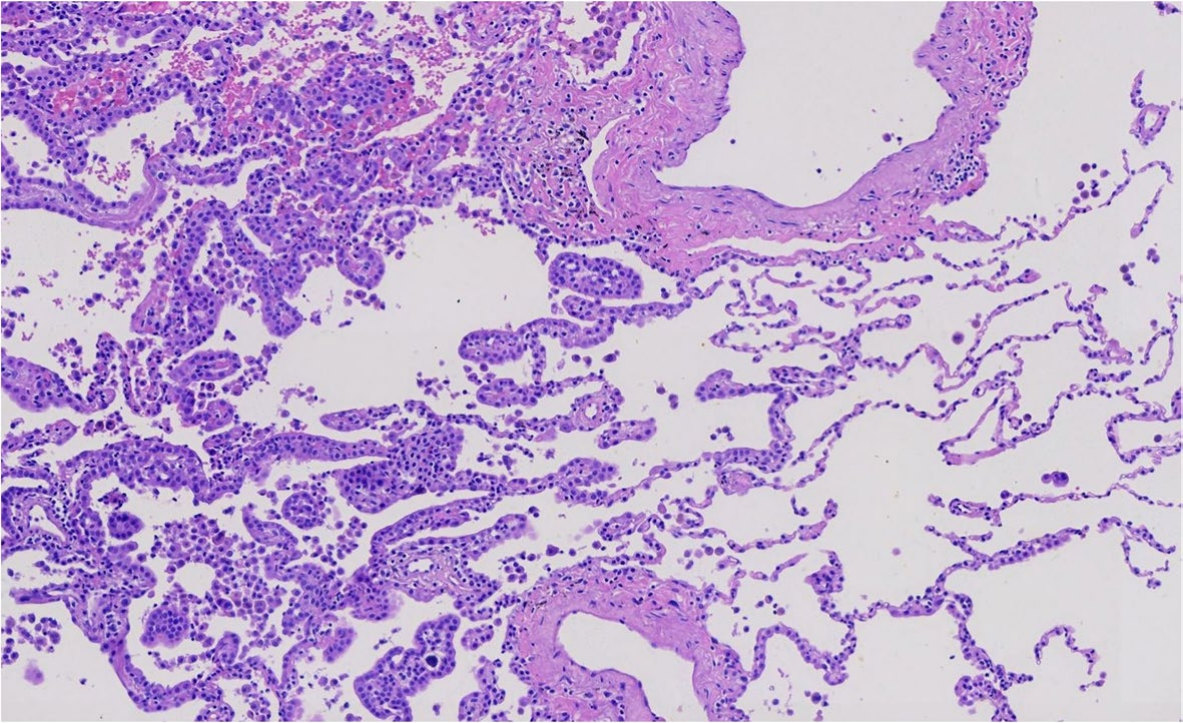
The alveolar structure exists, and the tumor grows mainly along the alveolar wall in an adherent manner

**Fig. 5:**
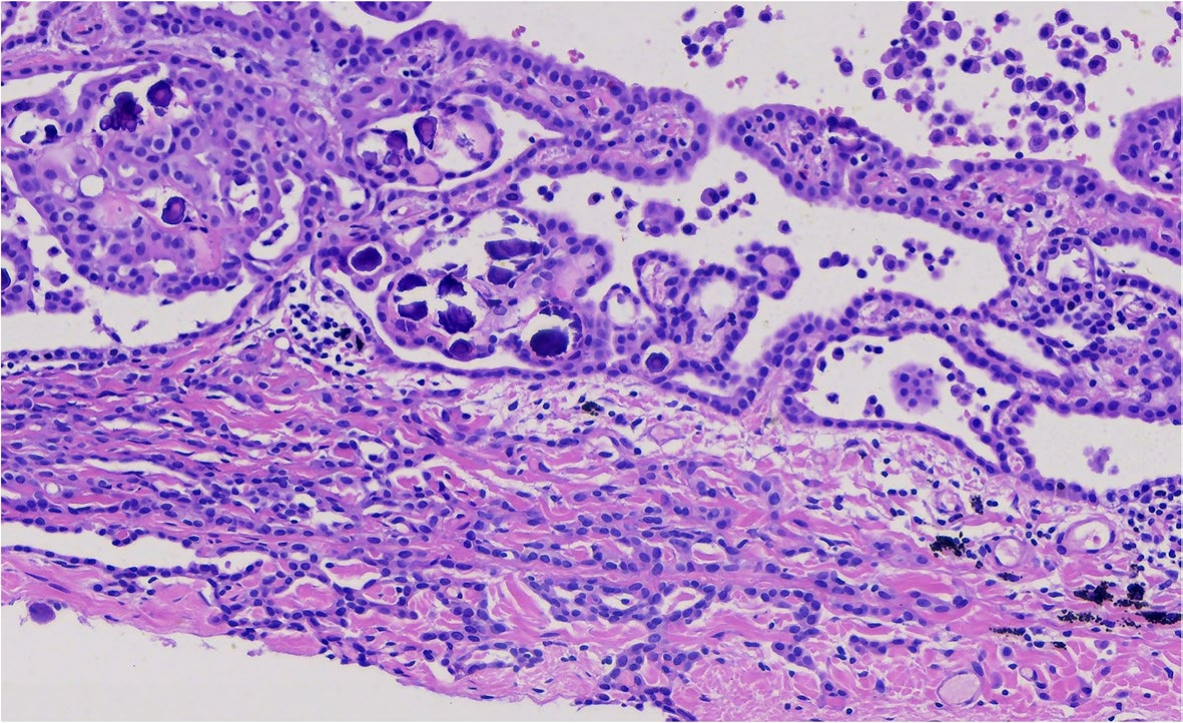
The tumor cells are medium, cuboid, tightly packed, and mildly shaped

**Fig. 6:**
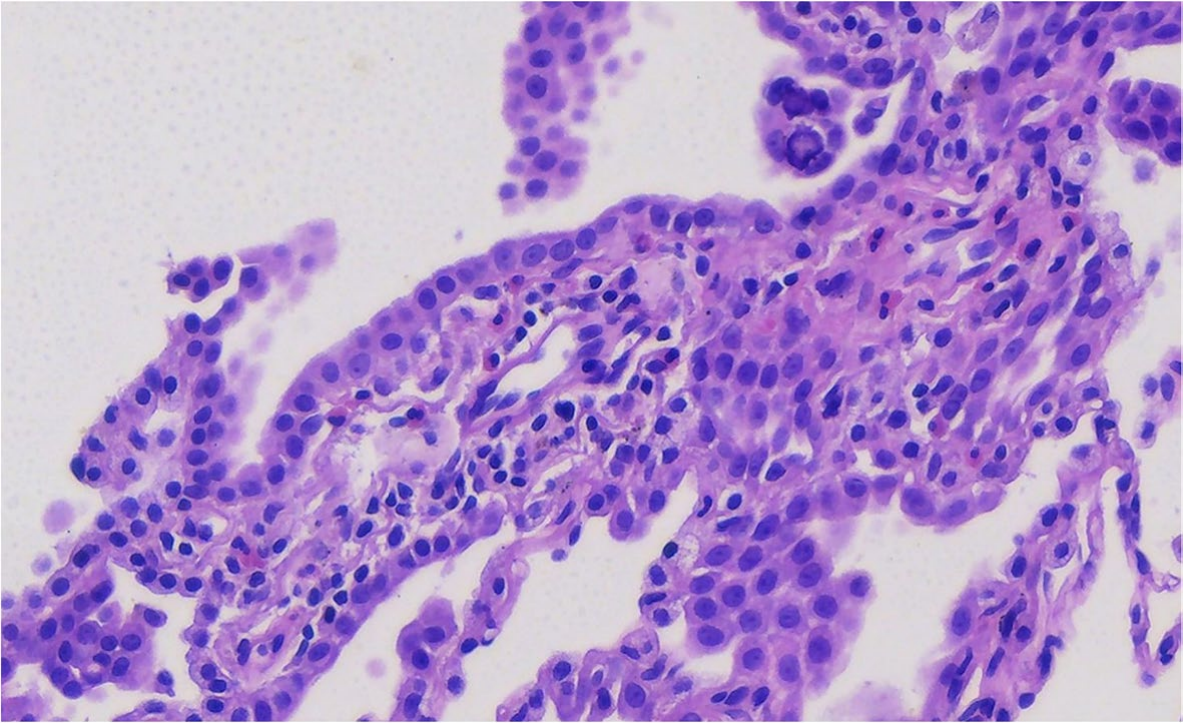
The tumor cells are medium, cuboid, tightly packed, and mildly shaped

Microscopically, the tumor invaded the lung and pleura with a boundary between the tumor and normal lung tissue, and there was no cell migration at the junction. The alveolar structure exists, and the tumor grows mainly along the alveolar wall in an adherent manner (Figs. 3 and 4). Some cells wrapped the fibrous vessel axis, and some were scattered in the alveolar cavity in small groups, showing papillary and micropapillary patterns. The tumor cells were medium, cuboid, tightly packed, and mildly shaped (Figs. 5 and 6). The chromatin was delicate, the nucleoli were rare, pathological mitotic figures were not found, and the amount of cytoplasm was moderate. The alveolar septa became thicker, fibroblasts proliferated in the interstitium, organization could be found, and there was a small amount of lymphocyte infiltration. Small number of lymphocytes, histiocytes and psammoma bodies were found in the alveolar spaces, without asbestos bodies or intravascular tumor thrombus. The tumors on the pleura were morphologically similar to lung tumors.

**Fig. 7:**
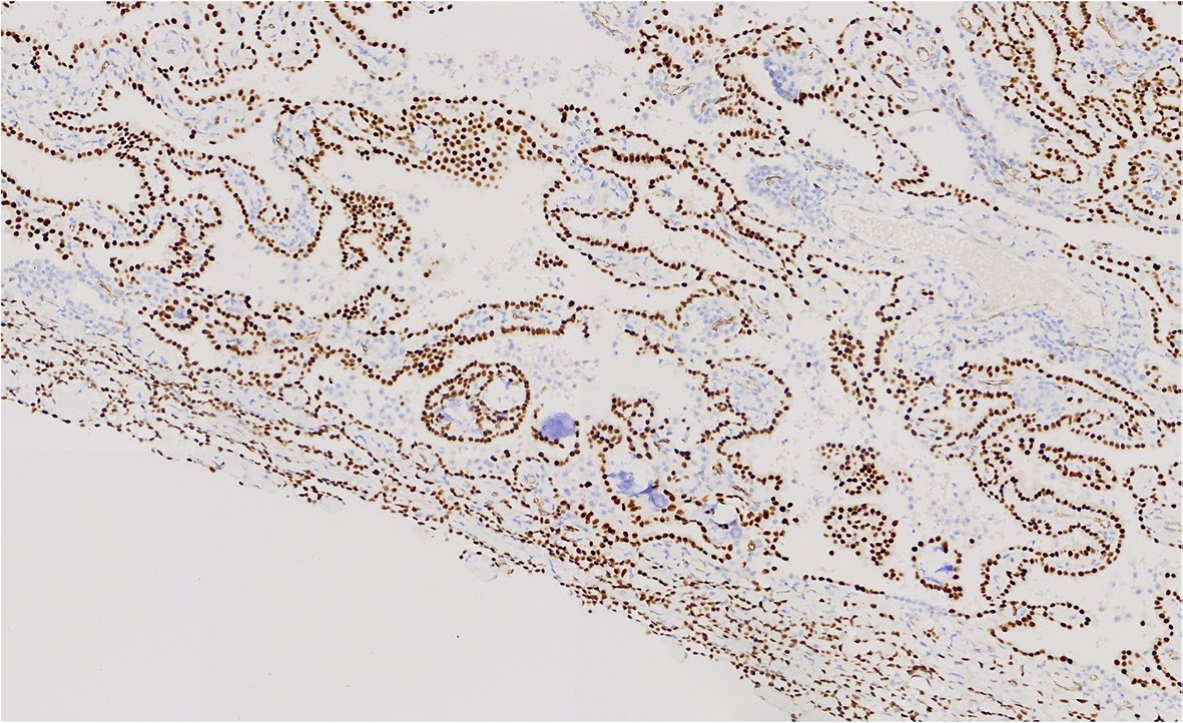
The tumor cells were positive for WT-1

**Fig. 8:**
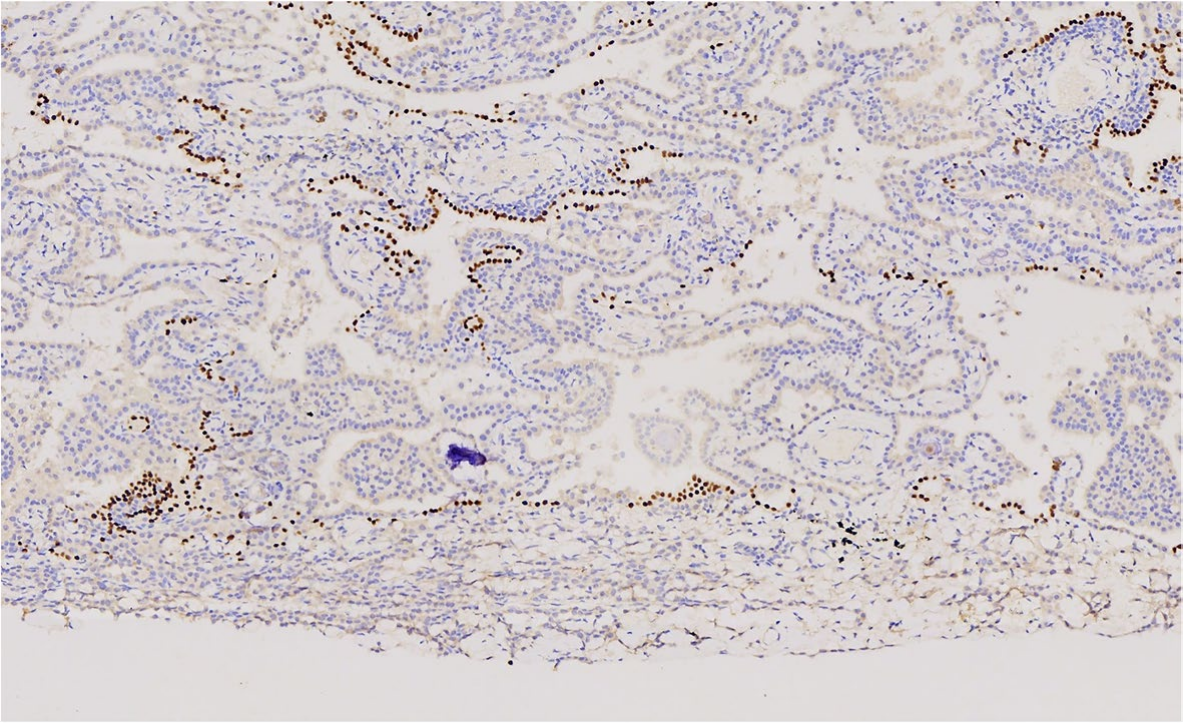
The tumor cells were negative for TTF-1

**Fig. 9:**
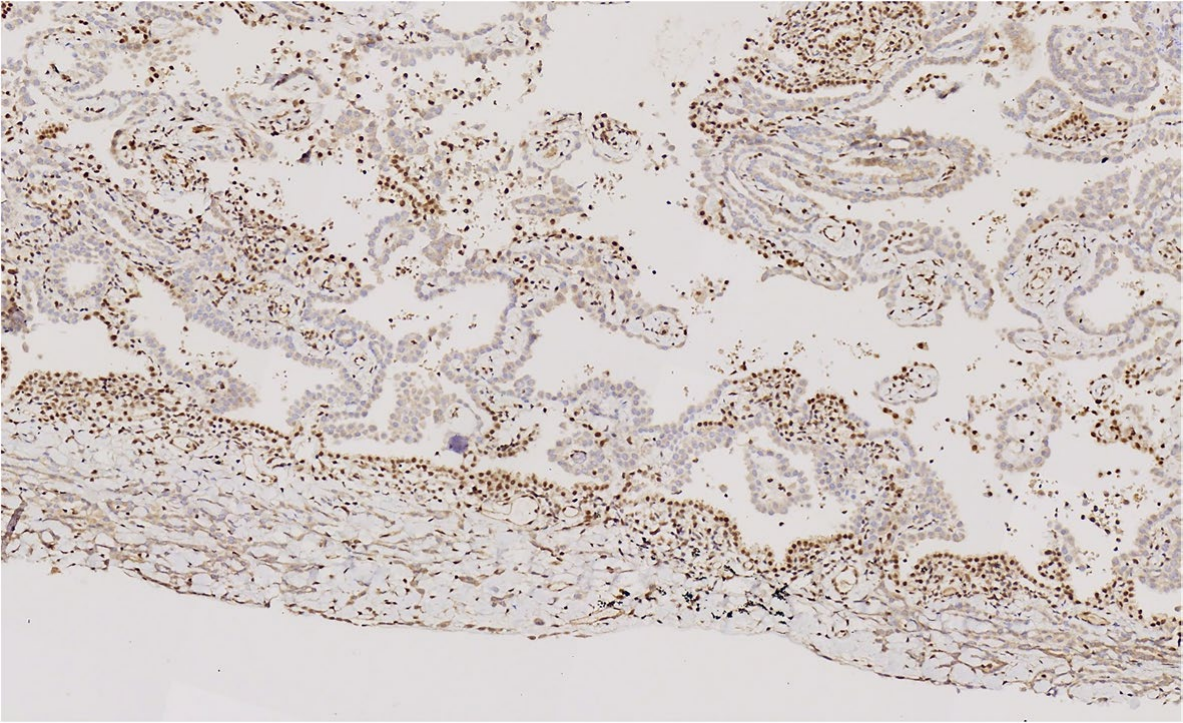
The tumor cells were negative for BAP-1

**Fig. 10:**
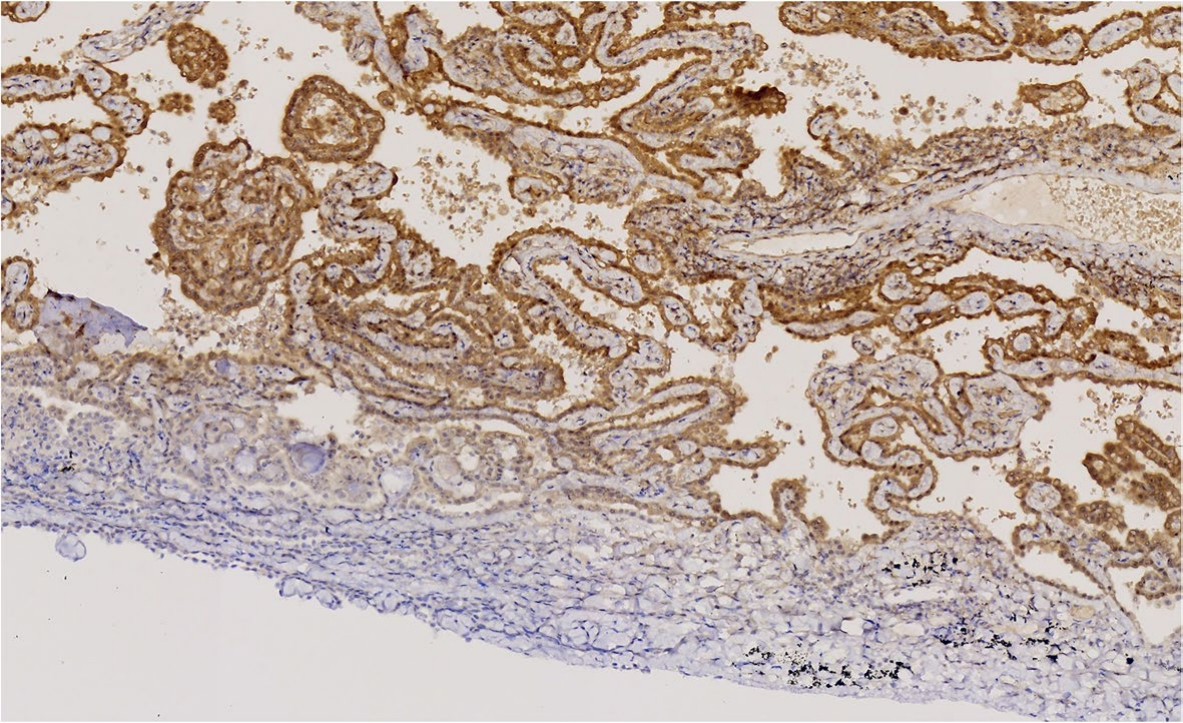
The tumor cells were positive for MTAP

Immunohistochemistry showed that the tumor cells were positive for CK, Claudin4 and mesothelial markers, such as WT-1, Calretinin, D2-40, and CK5/6. They were negative for alveolar epithelial markers, such as TTF-1, CEA, EMA, and CK7. BAP1 loss and MTAP expression were also observed (Figs. 7, 8, 9 and 10).

Fluorescence in situ hybridization ( FISH) showed a normal pattern of CDKN2A. No homozygous deletion was observed in the tumor cells.

Based on the patient’s contact history, imaging, histological morphology, immunohistochemical results, and FISH test results, the final diagnosis was DIM.

The patient received immunotherapy postoperatively and lost 20 kg of body weight within 9 months, and the general condition was poor.

## Discussion

DIM is a special growth pattern of PM. It is characterized by intrapulmonary growth, with or without pleural lesions or malignant pleural effusion, clinically and radiologically simulating interstitial lung disease. DIM is very rare. Only 16 cases of DIM have been reported in the English language, most of which are case reports. We collected these reported cases of DIM and summarized their clinical, imaging, and pathological features (Table 1).

**Table 1:** Clinical features of DIM

#	References	Case	Sex	Age (y)	Asbestos Exposure	Initial Symptoms	Thoracic Imaging Findings	Major Histologic Growth Patterns	FISH	Postoperative Treatment	Survival
1	Musk AW et al. 1991 [3]	1	M	44	Yes	Nasal congestion, LAD for 1 month	Bilateral military opacities; no pleural disease, effusions, or masses	Solid	—	Etoposide	12 weeks
2	Ohishi N et al. 1996 [4]	1	M	50	Yes	DOE for 1-week	right-sided massive pleural effusion and diffuse reticulonodular shadow in the left lung	Lepidic	—	a course of systemic chemotherapy with cisplatin and mitomycin	5 months
3	Wu et al. 1996 [5]	1	M	69	No	right hydropneumothorax and DOE	right hydropneumothorax as well as minimal pleural thickening in the right upper lobe	Lepidic	—	—	—
4	Heki U et al. 1999 [6]	1	F	59	—	Weight loss, malaise, cough, fever for 5 months	Irregular nodular opacities in RLL; no pleural disease or masses	—	—	—	—
5	Rossi et al. 2006 [7]	1	M	64	No	Progressive SOB, COPD	Right PTX; emphysema with RUL bullae; no pleural thickening or masses	Lepidic	—	—	—
6	Felner et al. 2006 [8]	1	M	72	No	pleuritic, nonexertional chest pain and dyspnea	unilateral pleural effusion and multiple bilateral nodules in the pleura and lung	lepidic	—	—	—
7	Larsen BT et al. 2013 [2]	5	M	53	No	Acute SOB, cough for several hours	Bilateral PTX; multiple blebs; irregular bilateral subpleural opacities with reticulation; small right pleural effusion; no masses	Lepidic, micropapillary	normal pattern in 3 cases of epithelioid DIM	cisplatin and pemetrexed Maintenance: pemetrexed alone	28 months
8			M	70	No	SOB for 6 months	Diffuse bilateral GGOs with reticulation; several small subpleural nodular densities; no pleural disease or effusions	Lepidic, acinar	—	Cisplatin and pemetrexed	3 weeks
9			M	55	No	Progressive SOB, cough, fatigue, diaphoresis for 3 months	Diffuse bilateral GGOs with focal peripheral reticulation; upper lobe consolidation; no pleural disease, effusions, or masses	DIP-like, micropapillary	—	No	21 months
10			M	56	No	DOE, intermittent cough	Diffuse bilateral opacities with course reticulation; no pleural disease, effusions, or masses	Pneumoconiosislike, acinar	—	Induction: carboplatin and pemetrexed Maintenance: erlotinib	4 weeks
11			M	81	Yes	Progressive SOB	Stable patchy irregular opacities, primarily right sided; right pleural effusion; no masses	Pneumoconiosislike, solid	—	No	11 months
12	Hasegawa M et al. 2014 [9]	1	M	75	No	No	multiple bilateral lung nodules,no dominant lung mass or pleural lesion	sarcomatoid	homozygous deletion	No	1 year
13	Hida Tomoyuki et al. 2015 [10]	1	M	67	Yes	SOB, fever, cough	diffuse granular shadowing in both lungs, right pleural effusion, and hilar and mediastinal lymphadenopathy	acinar,solid	homozygous deletion	two courses of chemotherapy (cisplatin + pemetrexed)	
14	Larsen BT et al. 2019 [11]	1	M	70	No	recurrent unilateral pleural effusion	nonspecific pulmonary infiltrates,no diffuse pleural thickening or pleural-based masses	Lepidic	heterozygous deletion in one case of epithelioid DIMM	—	—
15	Kumazawa M et al. 2019 [12]	1	M	69	Yes	dyspnea for 3 months	diffuse miliary nodules,right pleural effusion,pleural thickening	acinar,solid,papillary	—	systemic chemotherapy	69 days
16	Prisciandaro Elena et al. 2020 [13]	1	F	67	No	DOE, cough	multiple bilateral pulmonary nodules, left pleural effusion plus subaortic and subcarinal lymph nodes enlargement	micropapillary	—	Cisplatin and Pemetrexed	2 months
17	This study	1	M	59	Yes	Recurrent unilateral pleural effusion	right pleural effusion, bilateral pure ground glass opacity lesions	Lepidic	normal pattern	immunotherapy	—

Among the 17 patients with DIM, 88.2% (15/17) were male and 11.8% (2/17) were female, aged 44–81, with a median age of 67. Most of them occurred in older men with non-specific clinical symptoms, such as dyspnea, cough, and fever; 37.5% (6/17) of the patients had a history of asbestos exposure, and 5.9% (1/17) of the patients had received chemoradiotherapy due to a previous lymphoma.

The typical imaging manifestations of PM are diffuse pleural thickening and multiple nodules, often encapsulating without invading the lung tissue, whereas DIM is mostly bilateral diffuse reticular lesions or nodules, with or without pleural thickening or pleural effusion [1, 2]. Of all the DIM cases we reviewed, 70.6% (12/17) presented with bilateral lung lesions and 52.9% (9/17) showed pleural effusion (Table 1). The imaging of our case showed pure ground-glass opacity lesions in both lungs with pleural effusion.

DIM has a variety of histological forms that can mimic the shape of lung adenocarcinoma, including lepidic, acinar, papillary, micropapillary, solid, and complex glandular features. Multiple forms can appear locally in the same lesion (Table 1). Among the 17 cases of DIM, 47.1% (8/17) showed the main lepidic growth pattern, which was consistent with the imaging findings of ground-glass masses. This pattern was also present in our patient.

DIM mainly consists of mild cuboidal epithelioid tumor cells with a moderate amount of eosinophilic cytoplasm, slightly larger uniform nuclei, and slightly irregular nuclear membranes. The tumor cells may have nucleoli and rare mitotic figures (< 1 pcs/20HPF). Some tumor cells may be spindle-shaped, without obvious atypia [2]. Fibroblasts hyperplasia, lymphocyte infiltration, hyaline degeneration, and organization may occur in the tumor stroma. The alveolar cavity may be filled with lymphocytes and phagocytes. In some cases of DIM, the tumor of the pleura may connect with the tumor in the lung, and intravascular tumor thrombus or malignant pleural effusion can be found. The case we report has no obvious intravascular tumor thrombus whose cytopathological examination of pleural effusion shows small round tumor cells of medium size and no obvious atypia, and is easily misdiagnosed as micropapillary lung adenocarcinoma metastasis.

The cytopathological examination of pleural effusion in the DIM case we report showed small round monoclonal cells of medium size and no obvious atypia, which were confirmed to be mesothelial origin by cell block embedding and immunohistochemistry. However, this diagnosis was inconsistent with the imaging characteristics of pure ground glass nodules. Therefore, the patient underwent exploratory thoracotomy, and the postoperative pathological diagnosis was DIM. For such lesions with atypical cell morphology, the pathological diagnosis should be combined with imaging and clinical, and the joint diagnosis should be carried out by means of cell block embedding or immunohistochemistry of surgical specimens.

Using electron microscopy, the tumor cells of DIM confirm the differentiation of mesothelial cells, with a large number of surface slender microvilli [5, 8], well-structured desmosomes, and scattered intracytoplasmic tension filaments.

The immunohistochemical expression of DIM is similar to that of PM, which is positive for CK and mesothelial markers such as WT-1, calretinin, D2-40, and CK5/6, and negative for epithelial markers such as TTF-1, CEA, EMA, CK7, CK20, and Claudin-4. BAP1 loss and MTAP expression can also be observed.

DIM is associated with a poorer prognosis than PM. The median survival time of patients with PM who received combined therapy (surgery, radiotherapy, or chemotherapy) was 6–18 months, while the 1- and 5-year survival rates were approximately 33% and 5% [14, 15]. The median survival time of patients with DIM is five months, with a median survival time of 4 weeks in untreated patients and 5–12 months after treatment (surgery or chemotherapy) (Table 1). The number of cases collected is too small to describe the role of traditional PM treatments (surgery or chemotherapy) on the prognosis of DIM. Tumor volume is critical because it predicts OS and assesses treatment response [16]. The prognosis of DIM is worse than that of PM, which may be related to the diffuse growth of DIM tumors in the lung and their larger size compared with PM.

In 17 cases of DIM, some showed intravascular tumor thrombus. We speculate that the formation of DIM lung lesions may be caused by tumor cells transferring to the lung tissue through blood vessels and lymphatic vessels [10]. In our case, the tumor cells persisted in the pleura and lung tissue, and no obvious intravascular tumor thrombus was observed. We speculate that the pleural tumor cells invaded the underlying lung tissue and adhered to the alveolar wall, thus preserving the alveolar structure. However, the reason why DIM shows obvious intrapulmonary lesions but not pleural lesions remains to be explored.

Differential diagnosis of DIM: ① Pulmonary adenocarcinoma: Pulmonary adenocarcinoma is the most common type of lung cancer, and imaging can reveal single, multiple nodules, or diffuse lesions. The growth pattern of DIM closely simulates pulmonary adenocarcinoma. Immunohistochemical analysis of tumor cells is positive for epithelial-derived markers such as CK, alveolar epithelial markers such as TTF-1, NapsinA, and CK7, and negative for mesothelial cell markers such as WT-1, calretinin, D2-40, and CK5/6. BAP1 is often expressed. ②Localized mesothelioma (LM): LM is another rare type of mesothelioma that accounts for approximately 0.5% of all mesothelioma cases [14]. LM, which refers to well-demarcated local plasma membranous or subserosal masses rather than diffuse growths, is histologically and immunohistochemically identical to a mesothelioma. LM can be completely resected by surgery and has a better prognosis than DIM. ③Reactive mesothelial hyperplasia (RMH): RMH is the local proliferation of mesothelial cells caused by various factors and is a non-neoplastic lesion. Reactive mesothelial cells are mild with small atypia. Immunohistochemical expression of mesothelial markers such as WT-1 and calretinin, BAP1 expression, and negative CDKN2A FISH results support the diagnosis of RMH. ④Interstitial lung disease (ILD): ILD is a non-neoplastic disease that often presents diffuse lesions in both lungs with ill-defined borders. Histologically, diffuse inflammation or fibrosis of the pulmonary interstitium may occur, with reactive proliferation of alveolar epithelial cells. Immunohistochemically, the mesenchymal cells expressed vimentin, desmin, SMA, and other mesenchymal markers.

## Conclusion

DIM is a rare type of PM. Radiology mainly manifests as diffuse lesions or nodules in the lung, with or without pleural lesions or malignant pleural effusion. DIM histology closely simulates pulmonary adenocarcinoma, presenting also with different growth patterns such as lepidic, acinar, papillary, micropapillary, solid, and complex glandular, while the atypia of DIM tumor cells is less than that of pulmonary adenocarcinoma. The prognosis of DIM is poor and the effect of traditional PM therapies on the prognosis of DIM is unknown. Awareness of this rare disease is essential to avoid misdiagnosis and to prevent delays in treatment.
